# Siberian roe deer (*Capreolus pygargus*) as an intermediate host of *Taenia lynciscapreoli* in Kazakhstan: molecular evidence

**DOI:** 10.1016/j.ijppaw.2026.101292

**Published:** 2026-09-21

**Authors:** Vladimir Kiyan, Christian Bauer, Rabiga Uakhit, Kira Kiyan, Karina Jazina

**Affiliations:** a Laboratory of Biodiversity and Genetic Resources, National Center for Biotechnology, Astana, Kazakhstan; b Department of Veterinary Medicine, S. Seifullin Kazakh Agrotechnical University, Astana, Kazakhstan; c Institute of Parasitology, Justus Liebig University Giessen, Giessen, Germany; d Scientific Center for Biological Research, Astana, Kazakhstan

**Keywords:** *Taenia lynciscapreoli*, *Capreolus pygargus*, Siberian roe deer, *Lynx lynx*, Kazakhstan

## Abstract

*Taenia lynciscapreoli* was recently reported from Eurasian lynx (*Lynx lynx*) in northern Kazakhstan, but its intermediate host in the country remains unknown. The aim of this study was to investigate, using molecular methods, whether Siberian roe deer (*Capreolus pygargus*) serve as intermediate hosts of *T. lynciscapreoli* in Kazakhstan.

Fifteen Siberian roe deer were legally harvested in three hunting grounds in north-central and eastern Kazakhstan between September 2024 and December 2025. *Taenia* metacestodes were detected exclusively in the lungs of four animals, all from a single hunting ground. BLASTn analysis of partial mitochondrial *nad1* sequences revealed 100% identity with reference sequences of *T. lynciscapreoli*, confirming the molecular identificationof all metacestode isolates as *T. lynciscapreoli*.

Despite the limited number of animals examined and the restricted geographical coverage, these findings–together with the earlier detection of the adult parasite in a Eurasian lynx from the same area–provide molecular evidence that *C. pygargus* serves as an intermediate host for *T. lynciscapreoli* in Kazakhstan. This study extends the known geographic range of the species and contributes to a better understanding of its transmission ecology in Central Asia.

## Highlights

• *Capreolus pygargus* was investigated as an intermediate host of *Taenia lynciscapreoli* in Kazakhstan.
• *T. lynciscapreoli* metacestodes were detected in the lungs of four Siberian roe deer.
• Molecular identification was confirmed by partial mitochondrial *nad1* sequencing.
• BLASTn analysis showed 100% identity with reference *T. lynciscapreoli* sequences.
• The study provides molecular evidence for completion of the *T. lynciscapreoli* life cycle in Kazakhstan.

## Graphical abstract

**Figure:**
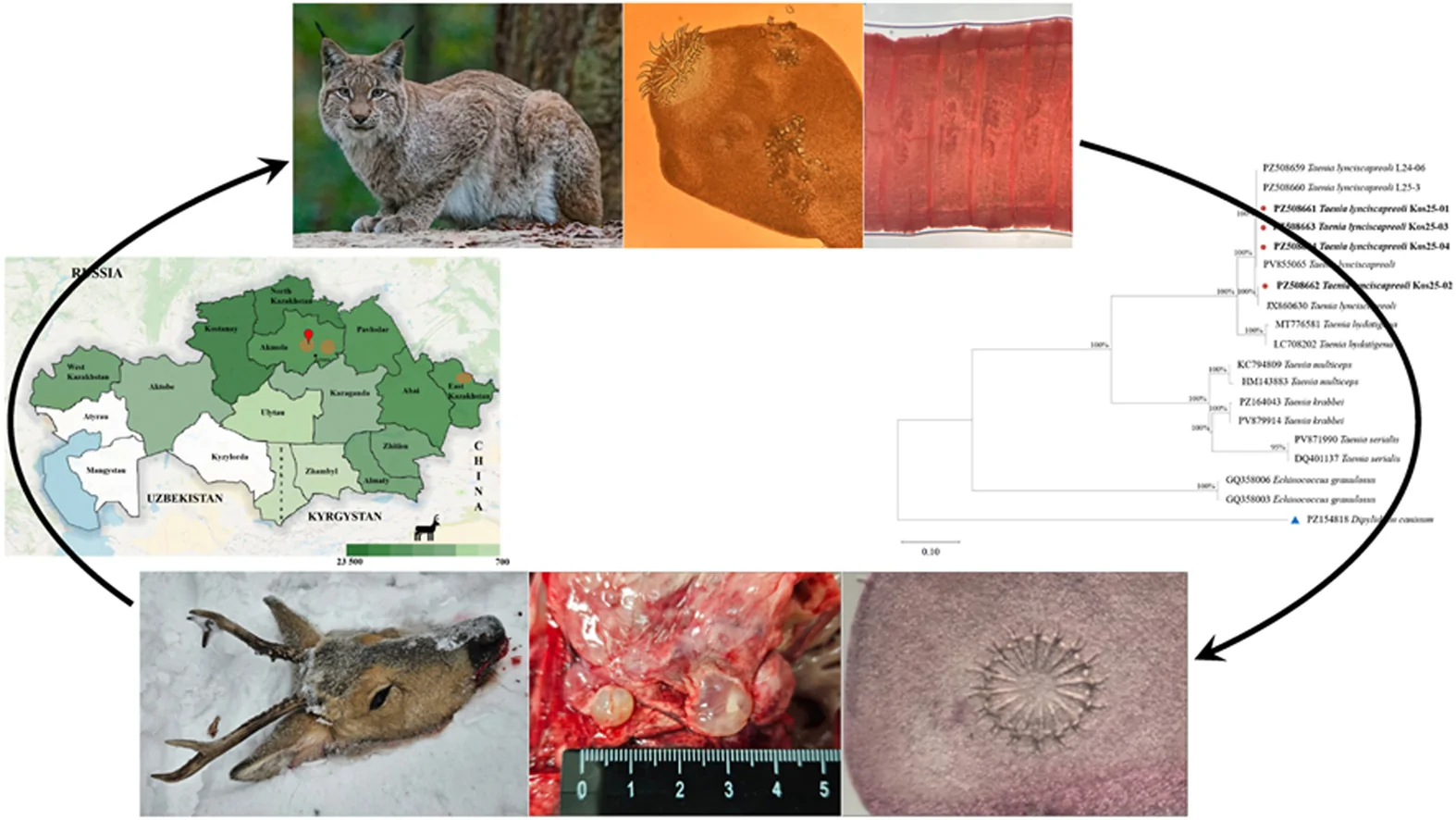

## 1 Introduction

*Taenia species* (Cestoda) are heteroxenous helminths whose transmission depends on predator–prey interactions. In wildlife ecosystems, terrestrial carnivores serve as definitive hosts, harboring adult tapeworms in the small intestine, whereas herbivorous or omnivorous mammals act as intermediate hosts, harboring the larval stages (metacestodes) in their tissues. *Taenia species* typically exhibit marked host specificity, with particular definitive hosts (e.g., canids, felids, or mustelids) and intermediate hosts (e.g., domestic ruminants, cervids, lagomorphs, or rodents). Their metacestodes also tend to develop in characteristic organs or tissues, including the omentum, subserosal tissue, musculature, brain, liver, and lungs. Consequently, the *Taenia fauna* of a definitive host is closely associated with the available prey community, whereas the metacestode fauna of intermediate hosts reflects the composition of the local predator community (Jones and Pybus, 2001).

For *Taenia lynciscapreoli*, the Eurasian lynx (*Lynx*) is the main definitive host, whereas the gray wolf (*Canis lupus*) has been reported as an occasional definitive host. Known intermediate hosts, in which metacestodes have been reported primarily from the lungs, include roe deer (*Capreolus*), Siberian roe deer (*Capreolus pygargus*), moose (*Alces alces*), and reindeer (*Rangifer tarandus*) (Haukisalmi et al., 2016; Kautto et al., 2022). Confirmed records of *T. lynciscapreoli* have been reported from Scandinavia (Haukisalmi et al., 2016; Kautto et al., 2022), several countries in Central and Southeastern Europe (Kołodziej-Sobocińska et al., 2018; Myczka et al., 2020; Lazár et al., 2026; Moraru et al., 2026; Drdlicek et al., 2026), as well as southwestern Siberia and the Russian Far East (Haukisalmi et al., 2016).

Recently, *T. lynciscapreoli* was reportedfrom Eurasian lynx in northern Kazakhstan (Lider et al., 2026), but its intermediate host in the country remained unknown. The Siberian roe deer, a known intermediate host in Siberia and the Russian Far East (Haukisalmi et al., 2016), is widely distributed in Kazakhstan (Argunov, 2025), and its population is estimated at approximately 115,250 individuals (Kiyan et al., 2024). It therefore represents a plausible intermediate host for *T. lynciscapreoli* in the country. The aim of this study was to identify the intermediate host of *T. lynciscapreoli* in Kazakhstan by molecular characterization of metacestodes collected from Siberian roe deer.

## 2 Material and methods

### 2.1 Animals, locations, and sampling

A total of 15 adult and subadult Siberian roe deer (*C. pygargus*) of both sexes were legally harvested during routine hunting activities by authorized hunters in three hunting grounds in north-central and eastern Kazakhstan (Fig. 1) between September 2024 and December 2025:

– Six animals were collected in the East Kazakhstan Region near the city of Altay (formerly Zyryanovsk), in the Bukhtarma River valley, an intermontane basin on the southwestern foothills of the Rudny Altai (49.738611°N, 84.271944°E).
– Five animals were collected in the Yereymentau District, Akmola Region, located at the northern edge of the Kazakh Uplands (Saryarka), an area characterized by rocky hills and ridges of the Yereymentau Mountains surrounded by open steppe (51.438518°N, 73.178516°E).
– Four animals were collected in the Akkol District, Akmola Region, situated in the northern part of the Saryarka Upland at the transition between the forest-steppe and dry-steppe zones (51.792955°N, 70.667700°E).

**Fig. 1:**
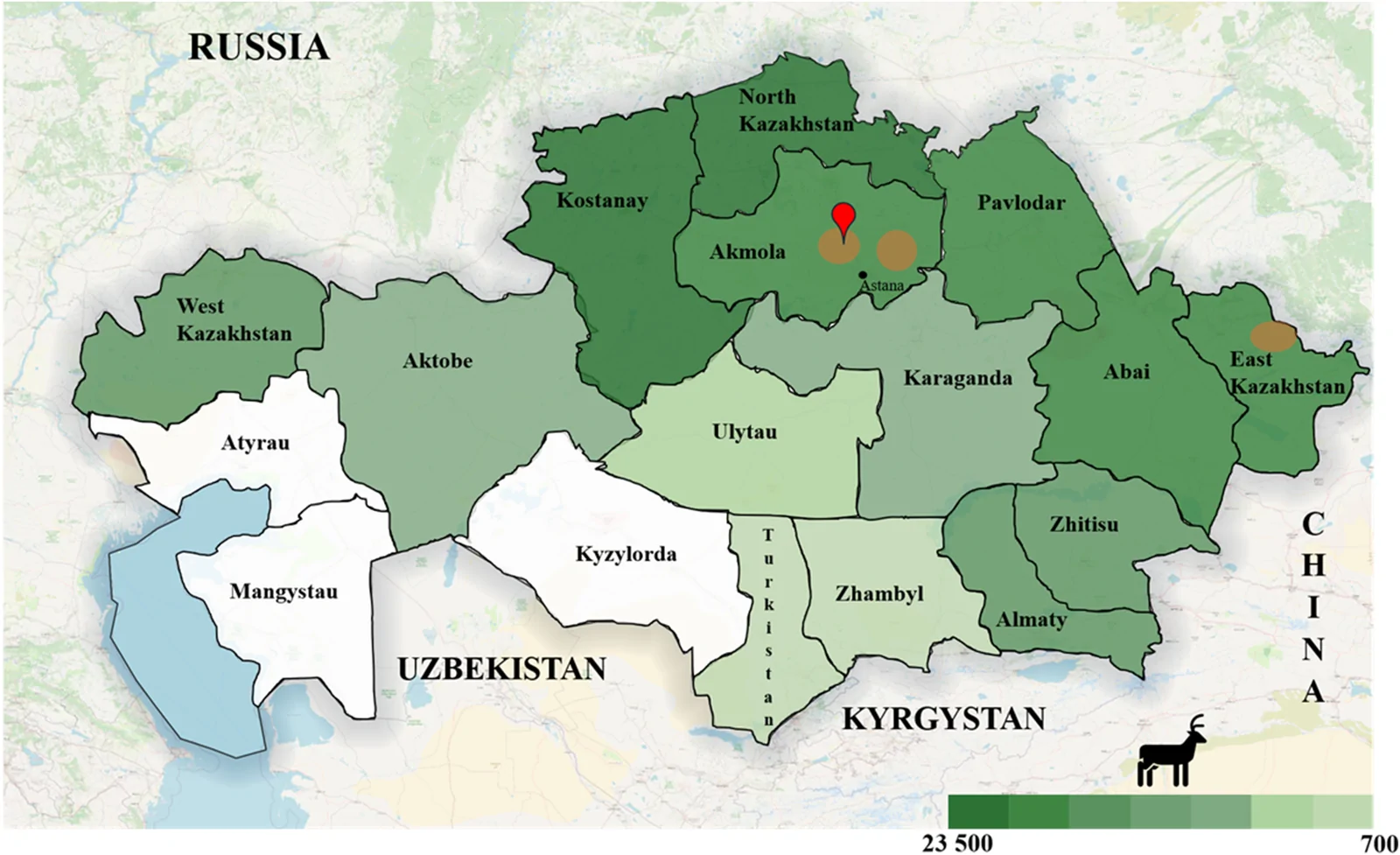
Map showing the estimated distribution of Siberian roe deer populations, sampling locations (shown in brown), and records of *Taenia lynciscapreoli* in Siberian roe deer (indicated by red pins) in Kazakhstan. The color scale, with increasingly darker shades of green, indicates the estimated Siberian roe deer population in each region, ranging from 700 to 23,500 individuals (Kiyan et al., 2024); regions without available population estimates are shown in white.

All roe deer exhibited morphological characteristics consistent with Siberian roe deer, *C. pygargus* (Argunov, 2025). To confirm species identity at the molecular level, one representative individual from each hunting ground was analyzed using polymerase chain reaction (PCR), followed by Sanger sequencing of a fragment of the mitochondrial D-loop/control region (Jäger et al., 1992; Randi et al., 1998). PCR amplification was performed using the primers L-Pro (5′-CGTCAGTCTCACCATCAACCCCCAAAGC-3′) and H-Phe (5′-GGGAGACTCATCTAGGCATTTTCAGTG-3′), which flank the mitochondrial control region. The resulting amplicons were purified and subjected to Sanger sequencing. All three individuals were genetically confirmed as *C. pygargus* (GenBank accession numbers PZ727150–PZ727152).

Following evisceration, the whole lungs, heart, liver, mesentery, and approximately 0.5 kg of thigh muscle were collected from each animal. Samples were transported under refrigeration to the Laboratory of Biodiversity and Genetic Resources, National Center for Biotechnology, Astana, Kazakhstan, where parasitological and molecular examinations were conducted within three days of collection.

### 2.2 Parasitological methods

In the laboratory, the lungs, liver, heart, mesentery, and thigh muscle samples were examined macroscopically for metacestodes, first externally and then following incision of the tissues, as described by Skrjabin (1928). Recovered metacestodes were isolated, counted, measured, washed in physiological saline, and preserved in 70% ethanol for subsequent morphological and molecular analyses.

### 2.3 DNA extraction

Genomic DNA was extracted from one representative metacestode isolated from each positive roe deer using the phenol–chloroform method. Approximately 20–30 mg of metacestode tissue was homogenized and incubated at 56°C for 2 h in lysis buffer (50 mM Tris-HCl, pH 8.0; 100 mM NaCl; 10 mM EDTA; 0.5% NP-40) supplemented with 5 μL of proteinase K (20 mg/mL). DNA was extracted and precipitated with ethanol, washed, air-dried, dissolved in 1× TE buffer, and stored at −20°C until further analysis (Sharma et al., 2023). DNA concentration and purity were assessed using a NanoDrop™ One spectrophotometer (Thermo Fisher Scientific, USA).

### 2.4 PCR analysis

Polymerase chain reaction was used to identify taeniid species using the primer pair JB11/JB12 targeting the mitochondrial *nad1* gene (Bowles and McManus, 1993); the estimated amplicon size was 450 bp. PCR amplification was performed using PCR Master Mix (2×) (Thermo Fisher Scientific, USA). PCR reactions were performed in a final volume of 25 μL with 10 pmol of each primer and 100 ng of genomic DNA as the template. PCR cycling conditions consisted of an initial denaturation at 95°C for 3 min, followed by 35 cycles of denaturation at 95°C for 60 s, annealing at 50°C for 60 s, and extension at 72°C for 60 s, with a final extension at 72°C for 5 min. Positive (*Taenia hydatigena* DNA) and negative (nuclease-free water) controls were included in each PCR run. Amplification products were visualized by agarose gel electrophoresis and documented using a GelDoc XR + transilluminator (Bio-Rad, USA).

### 2.5 Sequencing and phylogenetic analysis

All PCR-positive products were purified using the Quick PCR Purification Kit (QIAGEN, Germantown, MD, USA) according to the manufacturer's instructions, and then subjected to sequencing. Sequencing was performed using the BigDye Terminator v3.1 Cycle Sequencing Kit (Applied Biosystems, Foster City, CA, USA) according to the manufacturer's instructions on a 3730XL DNA Analyzer 96-Capillary Array (Thermo Fisher Scientific, Applied Biosystems, Foster City, CA, USA). Chromatograms were inspected manually, and low-quality regions were trimmed. The resulting *nad1* sequences were compared with sequences available in GenBank using BLASTn to confirm species identity. Sequences obtained in this study and reference sequences retrieved from GenBank were aligned using MUSCLE. Phylogenetic analysis was performed in MEGA11 (Tamura et al., 2021), the evolutionary history was inferred by using the Maximum Likelihood method and Tamura-Nei model. Initial tree for the heuristic search were obtained automatically by applying Neighbor-Join and BioNJ algorithms to a matrix of pairwise distances estimated using the Tamura-Nei model and then selecting the topology with superior log likelihood value. Discrete Gamma distribution was used to model evolutionary rate differences among sites (5 categories (+G, parameter = 0.4505)). This analysis involved 19 nucleotide sequences. The final dataset included 403 positions. *Dipylidium caninum* was used as the outgroup. Node support was evaluated by bootstrap analysis with 1000 replicates.

Nucleotide sequence data for the Siberian roe deer *T. lynciscapreoli* isolates obtained in this studywere deposited in the NCBI GenBank database under accession numbers PZ508661–PZ508664.

## 3 Results

Metacestodes were detected in 4 of 15 (27%) Siberian roe deer (1–4 years old; three males and one female; according to the hunting records). All positive animals were collected from a single hunting ground (Akkol District, Akmola Region; Fig. 1), where all four examined roe deer were infected (100%). The metacestodes were located exclusively in the lungs, occurring both subpleurally (Fig. 2A) and deep within the lung parenchyma (Fig. 2B). No metacestodes were detected in the liver, heart, or muscle tissue of any examined animal. The total number of metacestodes was 21, and infection intensity ranged from 2 to 8 (mean = 5.3). The metacestodes measured 0.6–1.5 cm in diameter (n = 14) and contained clear fluid and a whitish, invaginated protoscolex. Each evaginated protoscolex measured 3–4 cm in length (Fig. 2C) and possessed an armed rostellum with two concentric rows of large and small hooks, with 32–34 hooks in total (Fig. 2D). The location and morphology of the metacestodes were consistent with those described for *T. lynciscapreoli* (Haukisalmi et al., 2016; Myczka et al., 2020; Kautto et al., 2022).

**Fig. 2:**
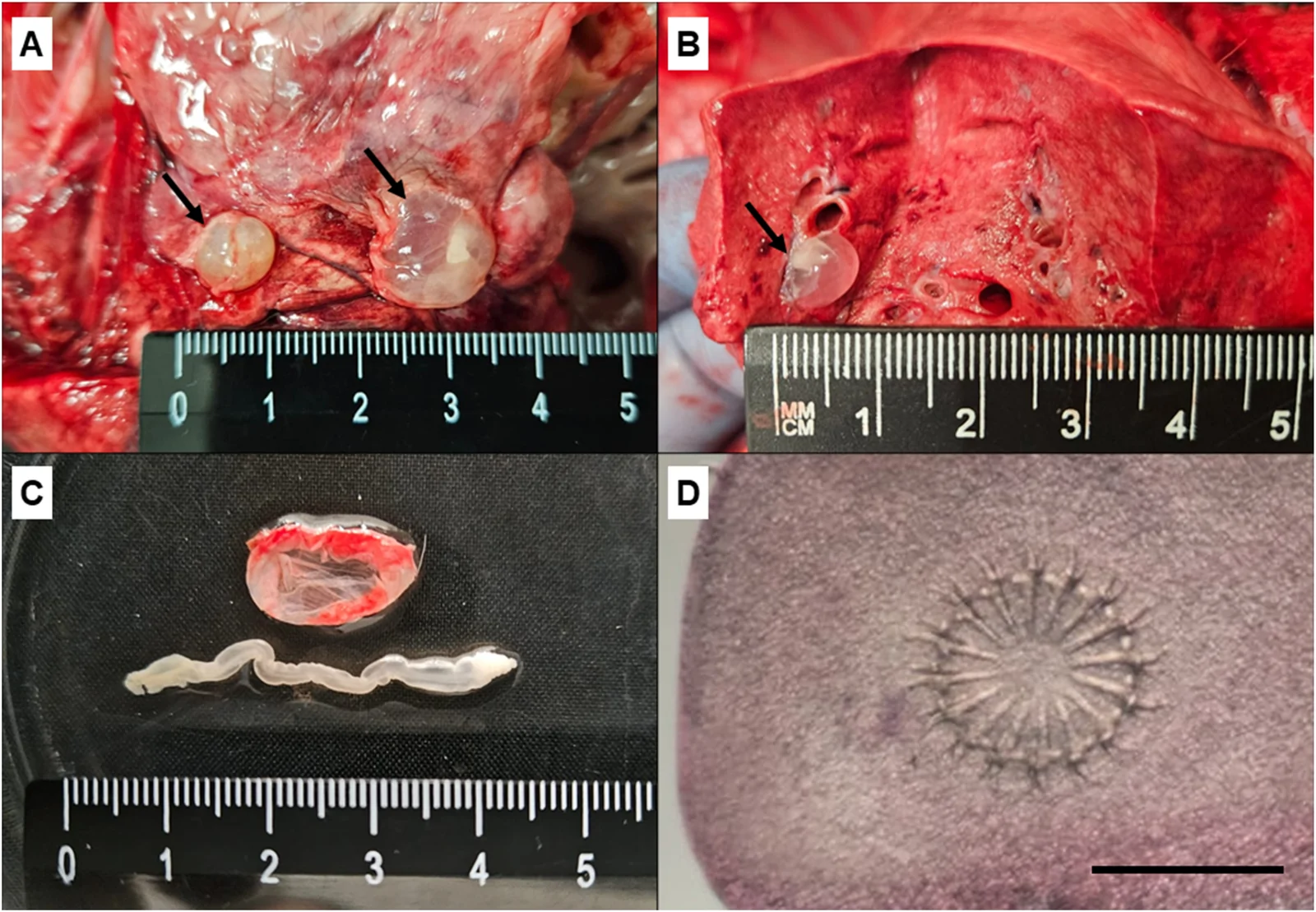
Morphological features of *Taenia lynciscapreoli* metacestodes recovered from the lungs of Siberian roe deer. (A, B) Metacestodes (arrows) located (A) subpleurally and (B) deep within the lung parenchyma adjacent to a bronchus. (C) Opened metacestode with an evaginated protoscolex. (D) Hook crown of a protoscolex showing two rows of large and small hooks. Scale bar = 500 μm.

Amplicons of approximately 420–450 bp were obtained from the four metacestode isolates. BLASTn analysis of the partial mitochondrial *nad1* gene sequences revealed 100% sequence identity with reference sequences of *T. lynciscapreoli* (JX860630 and PV855065). Phylogenetic analysis of partial mitochondrial *nad1* sequences placed all *T. lynciscapreoli* sequences from the present study, along with reference sequences PV855065 and JX860630, in a well-supported clade (100% bootstrap support) (Fig. 3). Within this clade, isolate PZ508662 grouped with JX860630 with 100% bootstrap support, whereas PZ508661, PZ508663 and PZ508664 grouped with the reference sequence PZ508659, PZ508660 (L24-06 and L25-3) lynx isolates previously reported from the same study area (Akkol District) (Lider et al., 2026) as well as with reference sequence PV855065 with 100% bootstrap support. The reference sequences of *T. hydatigena, T. multiceps, T. krabbei*, and *T. serialis* formed separate species-specific groups in the analysis. *Dipylidium caninum* was used as the outgroup. Overall, the phylogenetic placement of the study sequences supported their molecular identification as *T. lynciscapreoli*.

**Fig. 3:**
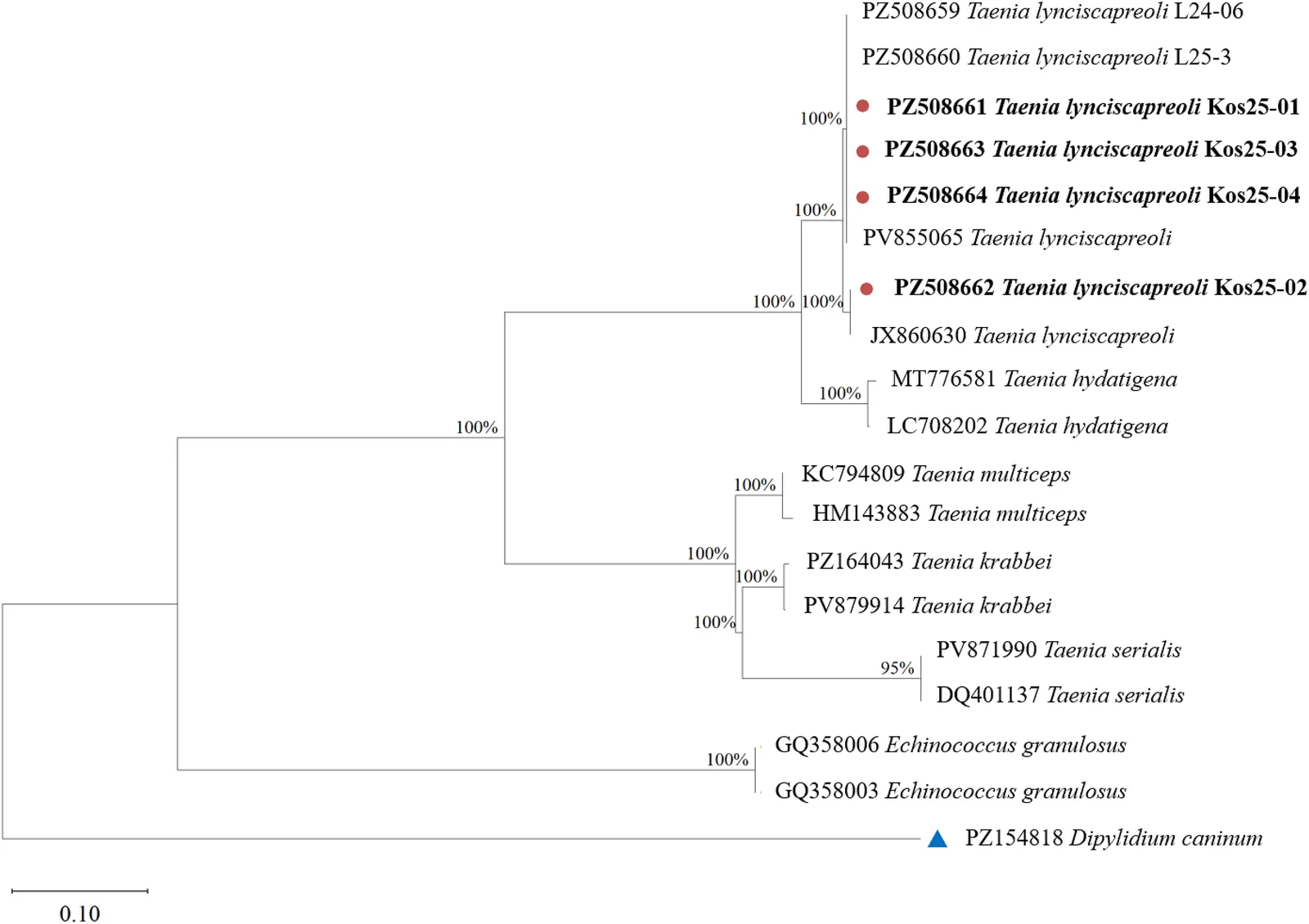
Phylogenetic tree of *Taenia lynciscapreoli* based on mitochondrial *nad1* gene sequences. Red circles indicate isolates obtained in this study. Reference sequences were retrieved from GenBank. The blue triangle indicates the outgroup (*Dipylidium caninum*). The scale bar represents 0.050 nucleotide substitutions per site. Node support was assessed using 1000 bootstrap replicates.

## 4 Discussion

Following the recent detection of *T. lynciscapreoli* in Eurasian lynx in northern Kazakhstan–the first record of this species in Central Asia (Lider et al., 2026) – the detection and molecular identification of its metacestodes in sympatric Siberian roe deer provide molecular evidence that *C. pygargus* serves as an intermediate host of *T. lynciscapreoli* in Kazakhstan. Together with the previous detection of the adult parasite in Eurasian lynx from the same area, these findings support the proposed linkage between the definitive and intermediate hosts of *T. lynciscapreoli* in the country.

Phylogenetic analysis of the mitochondrial *nad1* gene showed that all examined metacestode isolates belonged to *T. lynciscapreoli*, whereas *T. serialis*, *T. krabbei*, and *T. multiceps* formed separate clades (Fig. 3). The Siberian roe deer isolates (Kos25-01 to Kos25-04) clustered with the lynx isolates L24-06 and L25-3 previously obtained from the same study area (Lider et al., 2026), as well as with reference sequences of *T. lynciscapreoli* from Europe, including Finland (Lavikainen et al., 2013; Haukisalmi et al., 2016) and Romania (Moraru et al., 2026). The slightly divergent position of isolate Kos25-02 may reflect intraspecific sequence variation; however, its placement within the *T. lynciscapreoli* clade supports its assignment to this species. These findings further demonstrate the value of mitochondrial markers for species identification and differentiation of closely related taeniid cestodes (Lavikainen et al., 2008; Haukisalmi et al., 2016; Poon et al., 2017). Although a limitation of this study is the relatively short *nad1* fragment analyzed, which may provide limited phylogenetic resolution and restrict the inference of relationships among closely related *Taenia* lineages.

The hunting area in Akkol District, where *T. lynciscapreoli* was detected in all four Siberian roe deer examined in the present study and in three of six Eurasian lynx examined by Lider et al. (2026), is estimated to support more than 20 lynx and approximately 370 Siberian roe deer (Kiyan et al., 2024). One possible explanation is that the co-occurrence of these predator and prey species may facilitate the maintenance and transmission of *T. lynciscapreoli* within this predator–prey system, although the limited number of animals examined should be considered. This interpretation is consistent with previous reports of the parasite in Eurasian lynx and roe deer populations in Scandinavia and Poland (Haukisalmi et al., 2016; Myczka et al., 2020), as well as in Siberian roe deer in Siberia and the Russian Far East (Haukisalmi et al., 2016). The feeding ecology of the Eurasian lynx further supports this association, as roe deer and Siberian roe deer are important prey in areas with high ungulate densities (Sidorovich, 2022; Khorozyan and Heurich, 2023).

Several taeniid species with canids as definitive hosts (Jones and Pybus, 2001) have been reported from Siberian roe deer in Kazakhstan. These include *Echinococcus granulosus* (Baitursinov and Berkinbay, 2021), *T. hydatigena* and *T. krabbei*/*T. cervi* (Akhmetov, 2011; Baitursinov and Berkinbay, 2021), which were identified morphologically, and *T. multiceps* (Kiyan et al., 2025), identified molecularly. In the latter study, the host was reported as *C. capreolus* but was subsequently reassigned to *C. pygargus* based on mitochondrial DNA analysis (V. Kiyan, unpublished data). Both animals were also hunted in the Akmola Region, although at a hunting ground geographically distant from Akkol District.

It remains unclear why *T. lynciscapreoli* metacestodes were detected at only one of the three hunting grounds examined in the present study and were not detected in the animals analyzed by Kiyan et al. (2025). This spatial restriction is noteworthy given that Eurasian lynx from the neighboring regions of Karaganda and North Kazakhstan have also been reported to harbor this cestode (Lider et al., 2026). This pattern may reflect local variation in lynx abundance, roe deer density, prey availability, predator–prey encounter rates, or stochastic effects. However, these explanations remain tentative and should be treated with caution because of the small sample sizes and restricted geographical coverage. Larger-scalestudies involving more lynx and Siberian roe deer, as well as other potential intermediate hosts such as moose, are needed to clarify the distribution and transmission ecology of *T. lynciscapreoli* in Kazakhstan.

## 5 Conclusion

The molecular identification of *T. lynciscapreoli*metacestodes from *C. pygargus*, together with the previous detection of the adult parasites in Eurasian lynx from the same area, provides molecular evidence that *C. pygargus* serves as an intermediate host of *T. lynciscapreoli* in Kazakhstan. This study extends the known geographic range of the species and contributes to a better understanding of its transmission ecology in Central Asia.

## CRediT authorship contribution statement

**Vladimir Kiyan:** Writing – review & editing, Writing – original draft, Supervision, Investigation, Funding acquisition, Data curation, Conceptualization. **Christian Bauer:** Writing – review & editing, Writing – original draft, Investigation, Conceptualization. **Rabiga Uakhit:** Writing – original draft, Investigation, Data curation. **Kira Kiyan:** Investigation, Data curation. **Karina Jazina:** Investigation, Data curation.

## Data availability

Original data are available on reasonable request from the corresponding author.

## Ethics approval

The three-year study (2023–2025) was approved by the Local Ethics Committee of the National Center for Biotechnology (Protocol No. 1, 1 April 2022). All animals were legally harvested under the hunting regulations of the Republic of Kazakhstan, and samples were obtained from legally hunted animals; therefore, no animals were killed specifically for this study.

## Funding

The research was carried out with financial support from the Committee of Science of the Ministry of Science and Higher Education of the Republic of Kazakhstan within the framework of project AP19576421 for 2023–2025.

## Declaration of competing interest

The authors declare that they have no known competing financial interests or personal relationships that could have appeared to influence the work reported in this paper.
